# Exploration of the Mechanism of Salvianolic Acid for Injection Against Ischemic Stroke: A Research Based on Computational Prediction and Experimental Validation

**DOI:** 10.3389/fphar.2022.894427

**Published:** 2022-05-25

**Authors:** Xiaoqiang Li, Kaimin Guo, Ruili Zhang, Wenjia Wang, He Sun, Ernesto Yagüe, Yunhui Hu

**Affiliations:** ^1^ Cloudphar Pharmaceuticals Co., Ltd., Shenzhen, China; ^2^ College of Pharmacy, Haihe Education Park, Nankai University, Tianjin, China; ^3^ Tasly Pharmaceuticals Co., Ltd., Tianjin, China; ^4^ Division of Cancer, Imperial College Faculty of Medicine, Hammersmith Hospital Campus, London, United Kingdom

**Keywords:** salvianolic acid for injection, computational systems pharmacology, ischemic stroke, PTGS1, PTGS2

## Abstract

Ischemic stroke (IS) is an acute neurological injury that occurs when a vessel supplying blood to the brain is obstructed, which is a leading cause of death and disability. S*alvia miltiorrhiza* has been used in the treatment of cardiovascular and cerebrovascular diseases for over thousands of years due to its effect activating blood circulation and dissipating blood stasis. However, the herbal preparation is chemically complex and the diversity of potential targets makes difficult to determine its mechanism of action. To gain insight into its mechanism of action, we analyzed “Salvianolic acid for injection” (SAFI), a traditional Chinese herbal medicine with anti-IS effects, using computational systems pharmacology. The potential targets of SAFI, obtained from literature mining and database searches, were compared with IS-associated genes, giving 38 common genes that were related with pathways involved in inflammatory response. This suggests that SAFI might function as an anti-inflammatory agent. Two genes associated with inflammation (*PTGS1* and *PTGS2*), which were inhibited by SAFI, were preliminarily validated *in vitro*. The results showed that SAFI inhibited PTGS1 and PTGS2 activity in a dose-dependent manner and inhibited the production of prostaglandin E2 induced by lipopolysaccharide in RAW264.7 macrophages and BV-2 microglia. This approach reveals the possible pharmacological mechanism of SAFI acting on IS, and also provides a feasible way to elucidate the mechanism of traditional Chinese medicine (TCM).

## Introduction

Traditional Chinese medicine (TCM) has played a significant role in treatment of a large variety of diseases for thousands of years and is still widely used nowadays. TCM uses prescriptions which usually consist of different components, such as plants, animals or minerals, depending on the disease to treat. However, due to the presence of a large variety of chemical components in prescriptions, it is difficult to identify their active component, its mechanism of action and to establish clinical parameters in order to test their therapeutic efficacy. In addition, many prescriptions have more than one single active component, and these may act synergistically to produce therapeutic benefit. These impediments have long delayed the modernization of TCM (Zhang et al., 2019).

In the last years, the development of network pharmacology has made possible the exploration of mechanisms of action at the molecular level of many herbal preparations used in TCM. This has generated a new research paradigm for translating TCM from an experience-based medicine to an evidence-based medicine system (Li and Zhang, 2013). In addition, the combination of virtual screening technology, computational, experimental, and clinical research, are contributing to a new direction in the TCM network pharmacology research of the future (Jiao et al., 2021; Wang et al., 2021). However, most of current network propagation algorithms ignore the direction and mode between interacting proteins. As a consequence, they poorly reflect the effect of a drug on an entire network, requiring further improvement for their use in TCM network pharmacology (Zhao et al., 2019).

Ischemic stroke (IS) is an acute neurological injury caused by the narrowing or occlusion of the blood supply arteries of the brain, which is a major leading cause of death worldwide and the number one cause for acquired long-term disability, resulting in a global annual economic burden (Herpich and Rincon, 2020). Thrombolysis with thrombolytic agents, such as alteplase and urokinase, is generally the first choice for the treatment of ischemic stroke. However, due to the narrow therapeutic time window for thrombolysis treatment, a high number of patients miss the best time for treatment.

Salvianolic acid for injection (SAFI) is a TCM preparation, approved by the Chinese Food and Drug Administration since 2011, which is extracted from *Salvia miltiorrhiza* and undergoes further separation and purification steps using standard chemical procedures. Its main water-soluble components include salvianolic acid B, rosmarinic acid, lithospermic acid, salvianolic acid D, and salvianolic acid Y (Jing et al., 2013; Jing-yao et al., 2015; Lei et al., 2015; Li et al., 2016; Li et al., 2018). SAFI has anti-inflammatory, anti-oxidative stress, and anti-platelet aggregation effects. Clinical trials have shown the beneficial effects for ischemic stroke with no significant adverse effects on liver and renal function, and no significant risk of bleeding, indicating a good safety profile (Tang et al., 2015; Zhuang et al., 2017; Yang, 2020).

In order to investigate the molecular mechanism of SAFI, we first collected information on its major components through literature mining, and compared their similarities with those of approved IS therapeutic drugs (including anticoagulant, antiplatelet, neuroprotective, and lipid lowering drugs). Subsequently, SAFI targets, collected from extensive literature mining, and IS drugs targets were enriched for biological process analysis. Predicted SAFI inhibition and activation targets were extracted by signed random walk with restart (SRWR) method, and were enriched for biological process and signaling pathways. Potential important targets were found *via* comparison with disease genes of ischemic stroke. Differences and similarities between SAFI and the approved IS therapeutic drugs were also compared by signaling pathways enrichment analysis. In addition, the potential important targets of SAFI treatment for IS were validated by QSAR (quantitative structure-activity relationship) model. Finally, the key targets were validated experimentally using enzymatic and cell-based assays.

## Materials and Methods

**FIGURE 1:**
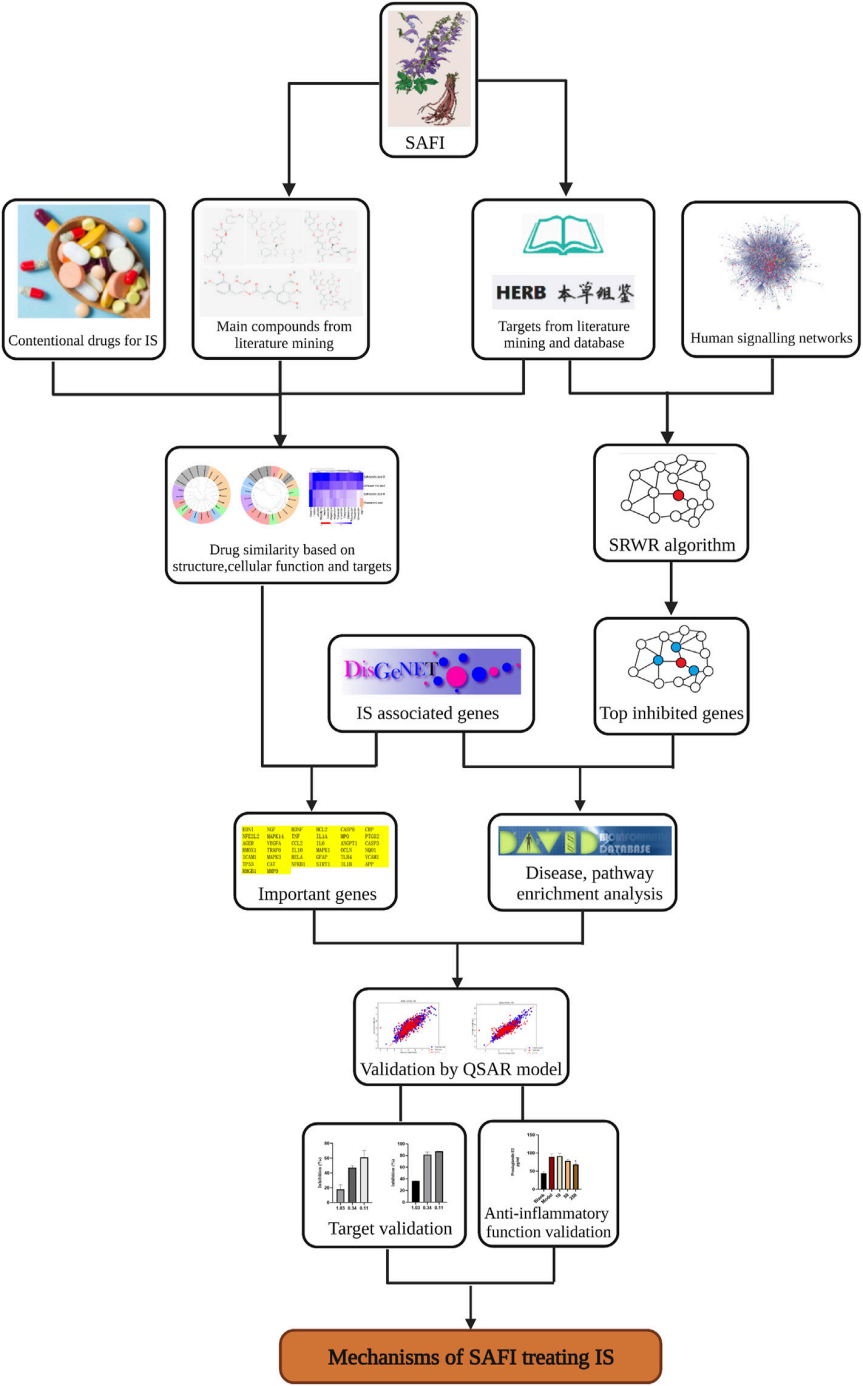
Workflow illustrating the steps used in the elucidation of SAFI mechanism of action in IS treatment. The workflow includes data collection, data analysis and experimental validations.

The whole workflow is illustrated in Figure 1.

### Collection of Information on SAFI Main Components

In order to collect information on SAFI main components, “salvianolic acid for injection” was used as the key word to obtain Chinese-language literature through CNKI (https://www.cnki.net/) and English-language literature through PubMed (https://pubmed.ncbi.nlm.nih.gov/). Then, the components contained in SAFI were collated manually and standardized through PubChem database (https://pubchem.ncbi.nlm.nih.gov/).

### SAFI’s Targets Obtained From the Literature and Database

In order to obtain SAFI target information, the term “salvianolic acid for injection” was used to obtain related literature through CNKI and PubMed databases.

The potential targets of five important SAFI components-salvianolic acid B, rosmarinic acid, lithospermic acid, salvianolic acid D, and salvianolic acid Y, were collected using HERB database (Fang et al., 2020).

### Collection of IS-Associated Genes, Anti-IS Drugs, and Their Targets

IS-associated genes were collected from the DisGeNET database (Piero et al., 2019) by searching the term “Ischemic stroke”. Differentially expressed genes in IS were obtained from GSE22255 dataset (downloaded from Gene Expression Omnibus (GEO), https://www.ncbi.nlm.nih.gov/geo/).

Anti-IS drugs collection were referred to “Chinese guidelines for diagnosis and treatment of acute ischemic stroke 2018” (Neurology and Society, 2018), and their targets were collected from the Drugbank database (Wishart et al., 2017).

### Correlation of Drug Targets and Disease Genes

To evaluate the efficacy of a drug on disease, the correlation of the drug targets and the genes associated with the disease from the perspective of network propagation was used (Yang et al., 2020). Briefly, the drug targets and disease genes were used as seed genes to run Random Walk with Restart (RWR) algorithm (Köhler et al., 2008) in the background network STRING, which was performed in R package dnet (version 1.1.7) with 0.75 of the restart probability. In this way, the influence score vector of the two sets of seed nodes on all nodes in the background network was obtained. Pearson correlation coefficient of the two score vectors (*Cor*) was then calculated, and *Z-score* was used to evaluate the significance of the correlation using the formula

$$z-\mathrm{score}=\frac{Cor-E\left(Cor\right)}{\delta \left(Cor\right)}$$

where *E(Cor)* and *δ(Cor)* are the mean and standard deviation of the Pearson correlation coefficients between the influence score vector of drug targets and those of 1000 groups of random contrast disease genes, each of which contained the same number of randomly selected proteins as the disease seed nodes.

### Drug Similarity Evaluation and Hierarchical Clustering Based on Chemical Structure, Comprehensive Targets and Cellular Function Fingerprints

The similarity of two drugs was evaluated based mainly on chemical structure, comprehensive targets or cellular function fingerprints. Drug clusters with similar features were obtained using hierarchical clustering.

The chemical structure similarity was measured based on the Morgan fingerprint using RDKit in python. Molecular fingerprints encode molecular structure in a series of binary digits (bits) that represent the presence or absence of particular substructures in the molecule. Comparing fingerprints enable us to determine the similarity between two molecules. Once SMILES strings are converted to scalar fingerprints, the Tanimoto coefficient was used as the similarity score to measure the absolute similarity between two molecules(Hakime et al., 2016).

The target similarity between two drugs was measured based on comprehensive targets of compounds. Two drugs acting on same targets could be considered to have the same effect. For multi-target drugs, drugs whose targets are very close in the PPI network show similar effects (Barabási et al., 2011). Here, the network proximity index proposed by Barabasi (Joerg Menche et al., 2015) was used to explore the similarity between two drugs. Briefly, the network proximity of drug target module A and drug-target module B is defined using the separation measure as follows:

$$S_{AB}=d_{AB}-\frac{d_{AA}+d_{BB}}{2}$$

which compares the mean shortest distance within the interactome of each targets module, ⟨*d*_*AA*_⟩ and ⟨*d*_*BB*_⟩, to the mean shortest distance ⟨*d*_*AB*_⟩ between targets module A and B. The smaller the *S*_*AB*_, the closer the topological distance between the two drugs, that is, the more similar the functions of the two drugs. BNet was used as the background human PPI network.

The cellular function fingerprint similarity of two drugs was measured based on a “compound-target-cellular function” heterogeneous network using the PathSim method (Yizhou Sun et al., 2011). As described in the literature (Fg et al., 2020), in the “compound-target-cellular function” heterogeneous network, the metapath “compound-target-cellular function target-compound” of two drugs was considered to describe the linkage between two drugs. In this instance, cellular function fingerprints of compounds were described using Gene Ontology (GO) biological processes term of targets of compounds. Under the metapath framework, PathSim was developed to find peer objects in the network and to measure the similarity of peer objects based on metapaths. The “compound-target-cellular function” heterogeneous network consists of drug-comprehensive target interactions and target-cellular function relations from the Gene Ontology database (The Gene Ontology, 2017).

Given a set of N compounds to be clustered, an N × N similarity matrix was generated. Finally, the similarity matrix was used to perform hierarchical cluster, which was executed by the R package hClust.

### Simulating the Propagation of the Impact of a Drug by Signed Random Walk With Restart (SRWR) and Evaluation Measure for the Impact of SAFI on the Network

The SRWR algorithm, which was proposed in the research of social networks for measuring trust and distrust in signed social networks (Jung et al., 2016), was used here to measure how the activation or inhibition of a seed node corresponding to a drug target, leading to the activation or inhibition of other nodes in the human signaling network.

As described in the literature (Zhao et al., 2019), suppose a signed random walker starts at one of the seed nodes s and walks on a signed network. The sign of the walker is either positive or negative, meaning that it exerts activation or inhibition to a node, respectively. At each step, the walker either moves to a randomly chosen neighbor u of the current node v or it jumps back to its starting seed node s and restarts its random walking. When the random walker goes through a negative edge, it changes its sign from positive to negative, or vice versa. Otherwise, it keeps its sign. Once the walker jumps back to its starting node, it regains its original sign. The SRWR can simulate the process that the activation or inhibition of a seed node propagates to other nodes in a signaling network.

The hypergeometric cumulative distribution was applied to quantitatively measure whether a gene set associated with IS (validation gene set) was more enriched with the genes significantly inhibited by SAFI (identified by SRWR) than would be expected by chance. We used IS-associated disease genes collected from DisGeNET as distinct validation gene sets. The detailed methods to define the *p*-value have been previously described (Zhao et al., 2019).

### Enrichment Analysis

For the purpose of exploring the biological function of genes correlated with IS, GO, Kyoto Encyclopedia of Gene and Genomes (KEGG) and tissue enrichment analysis were performed using DAVID. Significance of each term was assessed with a *p*-value, and a term with *p-value* <0.05 was considered being significant.

### Structure-Activity Relationship Between the Main Components of SAFI and IS-Associated Genes

The dataset of key common genes in IS and SAFI was obtained from the ChEMBL database (Anna et al., 2012), and processed in the Knime workflow. IC_50_ values in Schrödinger’s Ligprep module were converted to predicted IC_50_ values (PIC_50_). The QSAR model based on traditional methods was generated for key common genes through Schrodinger’s AutoQSAR module.

AutoQSAR is an automated machine learning application for building, validating, and generating quantitative structure-activity relationship (QSAR) models. The process of descriptor generation, feature selection, and creation of a large number of QSAR models has been automated into a single workflow in AutoQSAR (Dixon et al., 2016). These models are built using various machine learning methods (Bayes, KPLS, MLR, PCR, PLS, and RP), and each model is scored using a novel method, considering R^2^ and Q^2^ respectively (de Oliveira and Katekawa, 2018). AutoQSAR randomly selects a combination of training set and test set, and allocates data to the learning set according to the same pattern. Then, it calculates physicochemical and topological descriptors and 2D fingerprints (linear, dendritic, radial or MOLPRINT2D) through the Canvas module. These methods are used in combination with machine learning methods and can be used to build various predictive models.

### Target Validation *in vitro*

To evaluate the influence on PTGS1 and PTGS2 by SAFI and obtain IC_50_ values, Pharmaron (Beijing) was commissioned to perform *in vitro* PTGS1 and PTGS2 enzymatic assays. The detailed information about the assays, such as the reagents, instruments, assay procedure, data analysis, and calculation is presented in Supplementary Methods.

In order to validate the influence of SAFI on PTGS2 activity, RAW264.7 macrophages were plated in 96-well plate and pretreated with different concentrations of SAFI for 1 h. Then cells were stimulated by lipopolysaccharide (LPS, 0.5 μg/ml) for 18 h to induce PTGS2 expression. Similarly, BV-2 microglia were treated by LPS(0.1 μg/ml) and different concentrations of SAFI for 24 h to induce PTGS2 expression. The prostaglandin E2 content in supernatants, generated by cyclooxygenase 2 (COX2, encoded by PTGS2) in the conversion of arachidonic acid, was assayed with enzyme linked immunosorbent assay according to the protocol of manufacturer(JINGMEI II, Lot 202107).

### Statistical Analysis

Values are represented as mean ± SD. Statistical significance was determined by One-way ANOVA in GraphPad Prism 8. *p*-value <0.05 was considered statistically significant.

## Results

### Identification of SAFI Important Targets in the Treatment of IS

**FIGURE 2:**
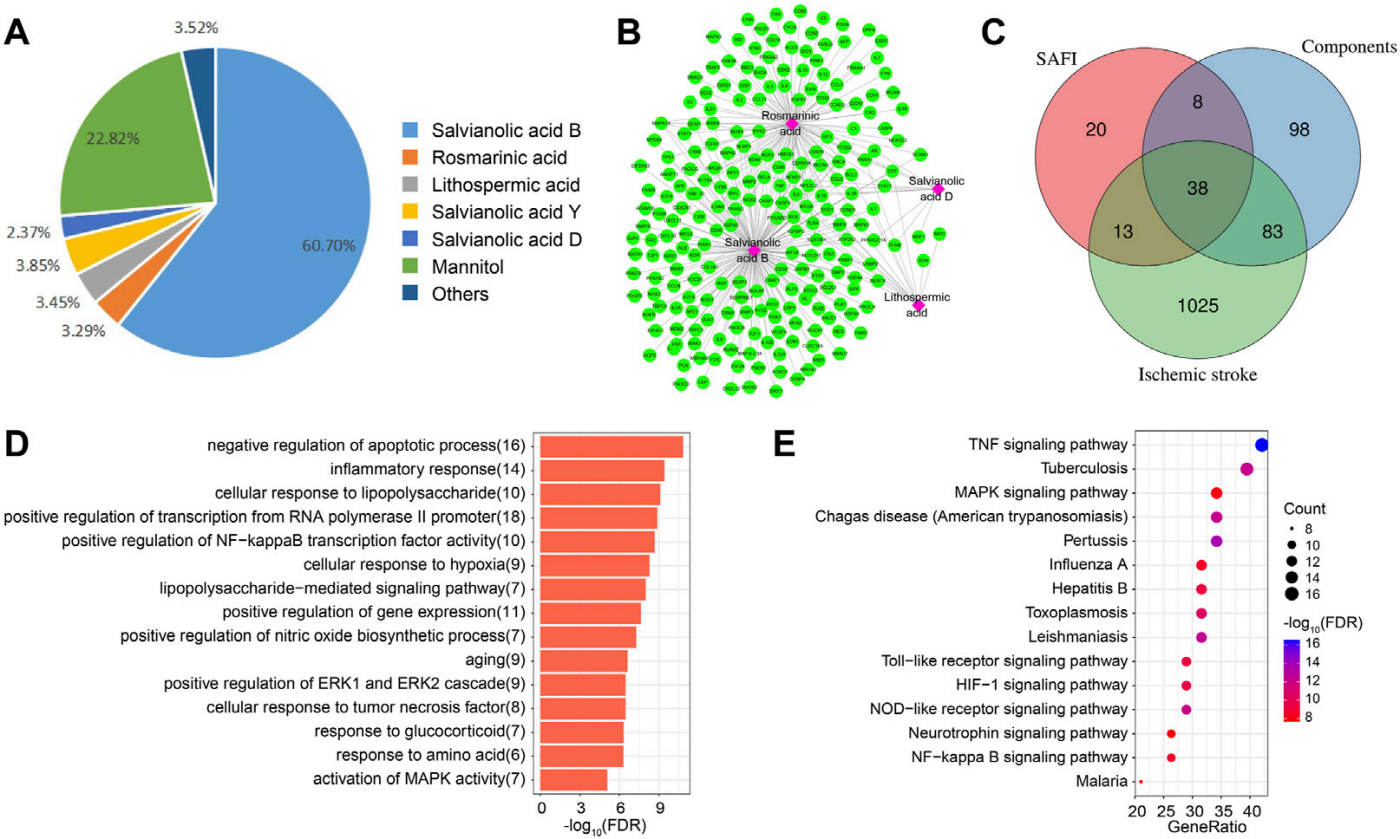
Potential targets of SAFI in the treatment of IS. **(A)** The main components and contents in SAFI collected from literature mining. It has to be noted that mannitol is used as pharmaceutic adjuvant in SAFI. **(B)** Potential targets of main components in SAFI. Pink boxes represent salvianolic acid B, rosmarinic acid, lithopermic acid, and salvianolic acid D, and green rounded boxes represent the corresponding targets of different components of SAFI collected from HERB database. **(C)** Overlaps between IS-associated genes from DisGeNET database, targets of SAFI collected from literature mining and targets of main components of SAFI (salvianolic acid B, rosmarinic acid, lithopermic acid, and salvianolic acid D) collected from literature mining and HERB database. **(D,E)** Functional annotations (biological process, **D**, and pathways, **E**) of 38 common genes obtained from overlaps by enrichment analysis.

In order to identify the important targets of SAFI in the treatment of IS, we first extracted its components through literature mining. It was found that salvianolic acid B, rosmarinic acid, lithospermic acid, salvianolic acid D, and salvianolic acid Y were major chemical components of SAFI, and four constituents present in the rat blood including salvianolic acid B, rosmarinic acid, lithospermic acid and salvianolic acid D were determined as effective Quality Markers of SAFI, identified by LC-MS/MS (Li et al., 2018) (Figure 2A; Table 1, Supplementary Figure S1). Subsequently, the targets of SAFI were collected from both literature mining and database searches. As a result, a total of 79 targets were collected from both CNKI and NCBI databases after searching with the keyword “SAFI” (Supplementary Table S1). Potential targets of SAFI’ main components were obtained from HERB database, and after manual removal of duplicates, 227 genes were obtained (Supplementary Table S2). The relationship between components and targets is shown in Figure 2B. Importantly, salvianolic acid B and rosmarinic acid possessed the most targets, whereas no targets were obtained from salvianolic acid Y after literature mining or database searches.

**TABLE 1:** Detailed information on main SAFI components.

Component	PubChem CID	InChI key	Canonical SMILES
Salvianolic acid B	11629084	SNKFFCBZYFGCQN-PDVBOLEISA-N	C1=CC(=C(C=C1CC(C(=O)O)OC(=O)C=CC2=C3C(C(OC3=C(C=C2)O)C4=CC(=C(C=C4)O)O)C(=O)OC(CC5=CC(=C(C=C5)O)O)C(=O)O)O)O
Salvianolic acid D	75412558	KFCMFABBVSIHTB-WUTVXBCWSA-N	C1=CC(=C(C=C1CC(C(=O)O)OC(=O)C=CC2=C(C(=C(C=C2)O)O)CC(=O)O)O)O
Salvianolic acid Y	97182154	SNKFFCBZYFGCQN-DUMGGQTMSA-N	C1=CC(=C(C=C1CC(C(=O)O)OC(=O)C=CC2=C3C(C(OC3=C(C=C2)O)C4=CC(=C(C=C4)O)O)C(=O)OC(CC5=CC(=C(C=C5)O)O)C(=O)O)O)O
Rosmarinic acid	5281792	DOUMFZQKYFQNTF-WUTVXBCWSA-N	C1=CC(=C(C=C1CC(C(=O)O)OC(=O)C=CC2=CC(=C(C=C2)O)O)O)O
Lithospermic acid	6441498	UJZQBMQZMKFSRV-RGKBJLTCSA-N	C1=CC(=C(C=C1CC(C(=O)O)OC(=O)C=CC2=C3C(C(OC3=C(C=C2)O)=CC(=C(C=C4)O)O)C(=O)O)O)O

We also collected the IS-associated disease genes from DisGeNET database, and obtained a total of 1159 disease genes (Supplementary Table S3). Figure 2C indicate the overlaps of SAFI and its main components and IS-associated genes. Intersection analysis highlighted 38 common genes (Supplementary Table S4) between IS, SAFI’s components and SAFI, representing 3.28% of IS genes, 48.1% of SAFI putative targets and 16.74% of main components datasets. The *PTGS2* gene was targeted by three main components of SAFI, salvianolic acid B, rosmarinic acid, and salvianolic acid D, suggesting an important role in the treatment of IS. Functional annotation of the 38 common genes was performed by enrichment analysis. As showed in Figure 2D, top GO terms were enriched in the BP category like *negative regulation of apoptotic process* and *inflammatory response*. Annotated pathways indicated that inflammatory associated pathways such as *TNF signaling pathway*, *HIF signaling pathway,* and *NF-kappa B signaling pathway* were significantly enriched in KEGG pathways (Figure 2E). In fact, apoptosis and inflammatory response are key characteristics of IS progression (Datta et al., 2020; Maida et al., 2020). In summary, we identified the putative target genes of SAFI correlated with IS and their association with inflammation.

### Drug Similarity Based on Structure, Function and Targets

**FIGURE 3:**
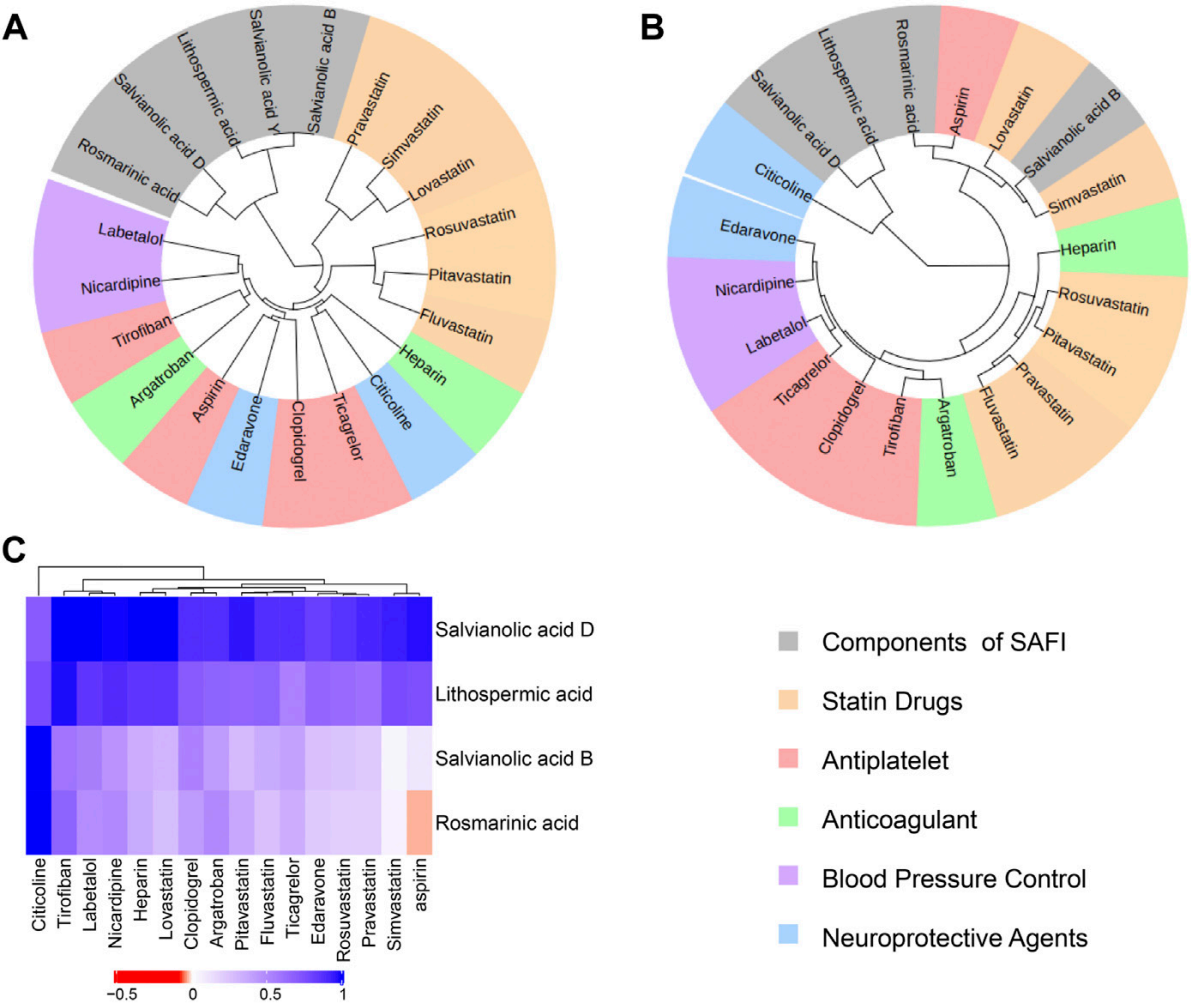
Drug similarity based on structures, functions and targets. Salvianolic acid B, rosmarinic acid, lithopermic acid, salvianolic acid Y, salvianolic acid D were compared with 16 recommended anti-IS drugs based on chemical structures **(A)**, functions **(B)** and targets **(C)**. In **(A)** and **(B)** panels, all compounds were divided into 6 subclusters with different background colors based on hierarchical clustering. Different types of approved drugs against IS are marked with different colors. In panel **(C)**, the depth of color indicates the network proximity (S_*AB*_) of the two drugs. The closer the color is getting to red, the smaller the S_*AB*_, and thus, the closer the topological distance between the two drugs, that is, the more similar the targets of the two drugs.

In order to investigate whether SAFI has a similar mechanism of action with current IS marketed drugs, we compared them using an unsupervised clustering evaluation method based on similarity. The “Chinese guidelines for diagnosis and treatment of acute ischemic stroke 2018” (neurology and society, 2018) recommends 16 drugs to treat IS, representing five different mechanisms of action, including antiplatelet (tirofiban, clopidogrel, ticagrelor, aspirin), anticoagulant (heparin, argatroban), antilipemic (lovastatin, pitavastatin, fluvastatin, rosuvastatin, pravastatin, simvastatin), antihypertensive (labetalol, nicardipine) and neoroprotectant (citicoline, edaravone). Next, the main compounds in SAFI were compared with the recommended anti-IS drugs based on similarity of chemical structure, functions and targets. The results showed that the main compounds of SAFI were not clustered with the five types of anti-IS drugs based on chemical structure (Figure 3A), indicating that the main compounds of SAFI do not share a similar structure with the recommended anti-IS drugs. However, hierarchical clustering of compounds showed that rosmarinic acid and salvianolic acid B were clustered with aspirin both based on functions and targets (Figure 3B,C), and salvianolic acid B was also clustered with simvastatin based on functions (Figure 3B). This indicates that SAFI may share similar effects with aspirin and simvastatin.

### Evaluation of Correlations Between SAFI and IS-Associated Genes

In order to evaluate and quantify the correlations between SAFI and IS-associated genes collected from DisGeNET, a parameter named *Z-score* was applied. *Z-score*, which provides a quantitative measure for the significance of the correlation between two genes, were set as greater than 3. *Z-score* indicated that the targets of both SAFI and its main components have a close link with IS-associated genes, especially salvianolic acid B (*Z-score* = 20.994) and rosmarinic acid (*Z-score* = 19.023), with a total of more than 200 IS-associated genes (Table 2).

**TABLE 2:** Correlation of targets for SAFI and its main components with IS-associated genes.

Drug	Targets^a^	Genes^b^	Original_coef^c^	Random_coef^d^	*Z score*
SAFI	79	1159	0.207	0.005	13.862
Salvianolic acid B	167	1159	0.322	0.014	20.994
Rosmarinic acid	108	1159	0.276	0.011	19.023
Lithospermic acid	15	1159	0.106	0.002	7.760
Salvianolic acid D	11	1159	0.084	0.003	6.071

a Number of drug targets.

b Number of IS-associated disease genes.

c Correlation coefficient between drug targets and IS-associated disease genes.

d Correlation coefficient of random extraction.

### Prediction and Identification of Genes Significantly Inhibited by SAFI Through SRWR Algorithm

Although it is known which targets obtained from literature mining are inhibited or activated by SAFI, the directionality of action of targets obtained from databases is unknown. The inhibition or activation of a target by a drug propagates to the other nodes in the human signaling network, and this can be measured by SRWR algorithm (Zhao et al., 2019), helping in the assessment on the potential impact of the drug. To construct the human signaling network, 7118 genes were used, and 4317 were used as the giant strongly connected component (GSC) in the background of the human signaling network. IS-associated genes, IS-GEO-UP genes and IS-GEO-down genes were used as the validation sets (Supplementary Tables S3, S5, S6). The method has been previously described (Zhao et al., 2019). We defined IS-associated genes significantly inhibited by SAFI as those with negative activation scores and with absolute values that ranked within the top 10% of all the nodes obtained with SRWR algorithm. In this way, the top 362 inhibited genes were identified. Table 3 shows the number of genes in common between the top inhibited genes by each drug and the three validation gene datasets. In most cases, the *p*-values were much smaller than 0.05, indicating the statistically significant enrichment of inhibited genes in the validation sets. The *p*-values in IS-GEO-UP dataset (the upregulated genes in GSE22255 dataset, Supplementary Table S5) were similar to those in IS disease genes (from DisGeNET), suggesting that SAFI may work as an inhibitor of IS-associated disease genes. It is worth mentioning that PTGS1 is in the top 10% inhibited by SAFI *via* SRWR algorithm, so it is also considered one of the most important targets of SAFI.

**TABLE 3:** Overlap numbers of top inhibited genes by each group of drugs with the three validation gene sets.

Drugs	Targets	Proportion in GSC	IS_disease_genes^a^		IS_GEO_UP^b^		IS_GEO_DOWN^c^	
			Genes	*p*-value	Genes	*p*-value	Genes	*p*-value
SAFI	Targets + −	71/78(91%)	122	0	17	0.001	7	0.357
Urokinase	(PLG PLAUR) +	2/2(100%)	6	0.256	0	NA	1	0.394
Aspirin	(PTGS1 PTGS2)-	2/2(100%)	101	1.274E-10	11	0.196	2	0.990
Heparin	(SERPINC1 F10)-	2/2(100%)	121	0	17	0.003	5	0.778
Nicardipine	(CACNA1C CACNB2 CACNA2D1 CACNA1D)-	4/4(100%)	76	0.001	11	0.189	5	0.772
Simvastatin	(ITGAL HDAC2)-	2/3(67%)	87	3.886E-06	14	0.032	7	0.455
Salvianolic acid B	Targets + −	138/167(83%)	114	0	15	0.006	5	0.689

a IS-disease genes set collected from DisGeNET.

b IS-GEO-UP set upregulated genes in GSE22255.

c IS-GEO-DOWN set downregulated genes in GSE22255.

To investigate whether the top inhibited genes by SAFI were expressed in IS target tissues, tissue enrichment analysis was conducted using DAVID. As shown in Table 4, a large number of the inhibited genes are expressed in whole brain and blood in the category of GNF_U133A_QUARTILE and UP_TISSUE, respectively (*p*-value < 0.05). These results suggest that SAFI could inhibit target tissue proteins to exert its anti-IS effects.

**TABLE 4:** Tissue enrichment analysis of the top inhibited genes by SAFI *via* SRWR algorirthm.

Category	Term	Count	Percent (%)	*p*-value	Benjamini
GNF_U133A_QUARTILE	Whole Brain_3rd	188	52.4	2.7E-22	2.1E-20
GNF_U133A_QUARTILE	Globuspallidus_3rd	117	32.6	3.7E-14	1.4E-12
GNF_U133A_QUARTILE	Medulla Oblongata_3rd	161	44.8	9.6E-14	2.5E-12
GNF_U133A_QUARTILE	LymphomaburkittsDaudi_3rd	107	29.8	4.9E-11	9.5E-10
GNF_U133A_QUARTILE	Trachea_3rd	106	29.5	1.5E-7	2.4E-6
UP_TISSUE	Blood	43	12.0	2.9E-10	6.0E-8
UP_TISSUE	Spleen	47	13.1	8.6E-10	6.0E-8
UP_TISSUE	Platelet	36	10.0	9.1E-10	6.0E-8
UP_TISSUE	T-cell	27	7.5	1.1E-9	6.0E-8
UP_TISSUE	Leukocyte	15	4.2	1.3E-6	6.0E-5
UP_TISSUE	Pancreas	44	12.3	2.6E-6	9.9E-5
UP_TISSUE	Placenta	99	27.6	1.8E-5	5.7E-4
UP_TISSUE	Neutrophil	6	1.7	3.4E-5	9.1E-4

For the 362 top inhibited genes predicted by SRWR algorithm, DAVID Functional Annotation Clustering tool was used to conduct the functional annotation from GO and KEGG analysis. The results showed that the top inhibited genes are related with processes such as *inflammatory response*, *platelet activation* and *cell adhesion*, which are all anti-IS associated process. KEGG enrichment analysis indicated that the top inhibited genes are involved in PI3K-Akt, TNF, NF-kappa B, and HIF signaling pathways, among others, which are associated with inflammatory response (Du et al., 2020; Minghua et al., 2021); Supplementary Figure S2). These results indicate that SAFI significantly inhibits a large number of genes associated with IS and inflammation, further validating its effect on IS.

### Prediction of Binding Activity Between SAFI and Identified Targets

**FIGURE 4:**
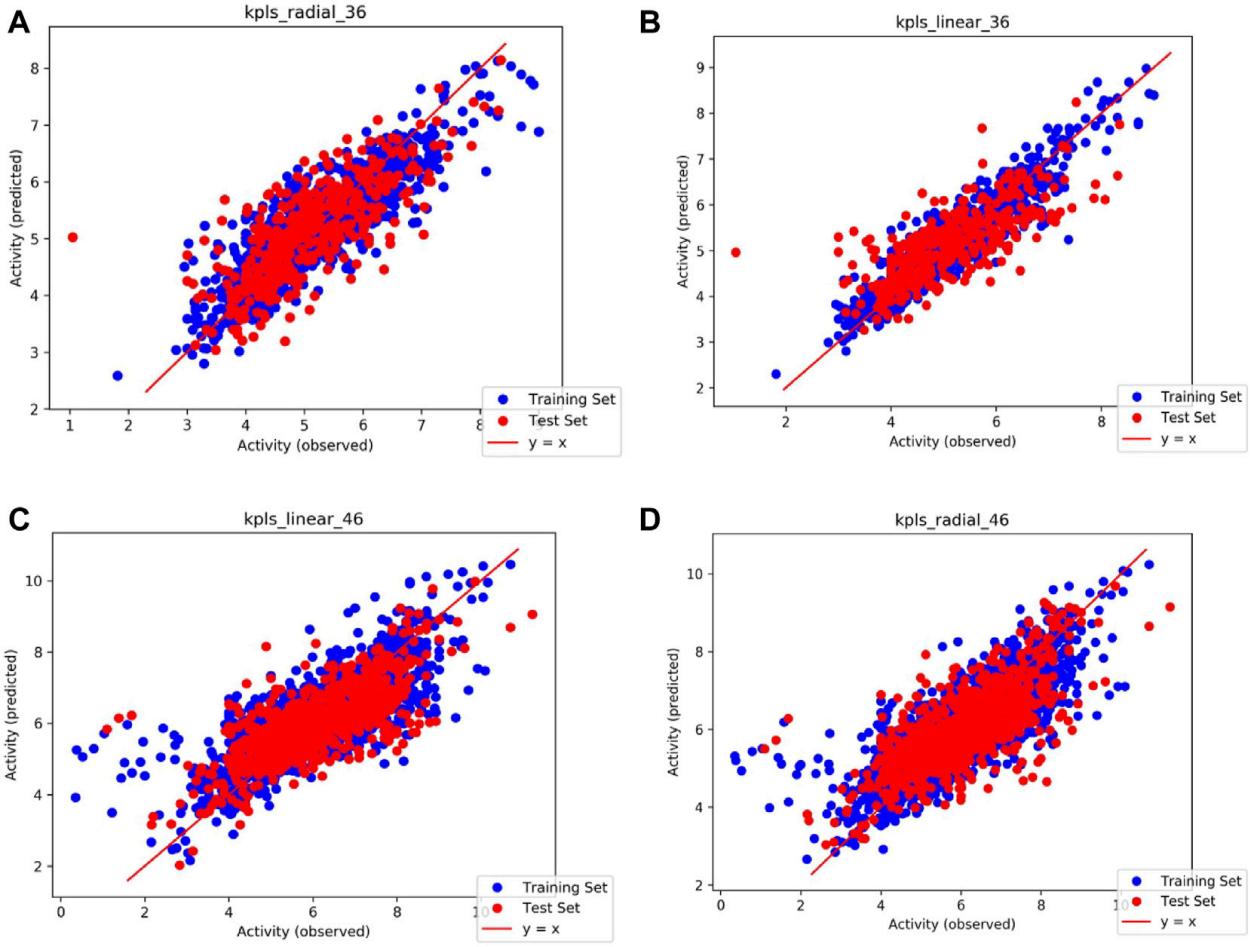
Scatter plots illustrating the performance of the QSAR model in predicting the experimental binding affinity of the learning set. **(A)** Model code: kpls_radial_36, generated by KPLS fitting radical features, using the 36th split of the learning set into test and training sets; **(B)** Model code: kpls_linear_36, generated by KPLS fitting linear features, using the 36th split of the learning set into test and training sets; **(C)** Model code: kpls_linear_46, generated by KPLS fitting linear features, using the 46th split of the learning set into test and training sets; **(D)** Model code: kpls_radial_46, generated by KPLS fitting radial features, using the 46th split of the learning set into test and training sets.

Based on the intersection analysis and SRWR results, *PTGS1* and *PTGS2* show big potential as important genes regulated by SAFI. We predicted the potential binding abilities between SAFI/main components and *PTGS1/2* using the QSAR model. The *PTGS1/2* dataset was downloaded from the ChEMBL database and processed in the Knime workflow. We prepared molecules with IC_50_ values in Schrödinger’s Ligprep module, and converted IC_50_ values to PIC_50_ values. The QSAR model based on traditional methods was generated for *PTGS1/2* through Schrodinger’s AutoQSAR module. R^2^ (correlation coefficient) and Q^2^ (cross validation coefficient) represent the availability of the model (the better model the closer to 1). By default, the dataset was divided into a 75% training set (*PTGS1*: 1217 molecules, *PTGS2*: 2634 molecules) and a 25% test set (*PTGS1*: 406 molecules, *PTGS2*: 879 molecules). We used two models with Q^2^ > 0.5 in top10 to predict the activity of the compound on the target. The model score of *PTGS1/2* with predictive ability is shown in Table 5, and the scatter plot in Figure 4 shows the performance of the model in predicting the experimental binding affinity of the learning set. We also validated the model with positive molecules, and the results are shown in Table 6. The prediction results showed that our model can identify molecules that are active to the corresponding target, and the difference in activity is no more than an order of magnitude. The activity values of SAFI predicted by the QSAR model are shown in Table 7. The results indicated that salvianolic acid B, lithopermic acid, salvianolic acid Y, and salvianolic acid D may possess inhibitory effect on *PTGS1* and *PTGS2* like two anti-IS drugs, aspirin and NS-398.

**TABLE 5:** Performance index of QSAR model of PTGS1/2.

Model name	Model code	R^2^	Q^2^
PTGS1	kpls_radial_36	0.7720	0.5850
	kpls_linear_36	0.8867	0.5532
PTGS2	kpls_linear_46	0.6367	0.5193
	kpls_radial_46	0.6633	0.5135

**TABLE 6:** The PIC50 value of the positive compound predicted by the QSAR model of PTGS1/2.

Drug	PTGS1	PTGS2	PIC_50_ (μM)	Actual activity value	Actual IC_50_ (μM) of drugs
Aspirin	3.787	—	163.305	4.557	27.750
SC-560	8.098	—	0.008	8.046	0.009
Celecoxib	—	7.017	0.096	7.398	0.04
Valdecoxib	—	6.552	0.281	8.301	0.005
NS-398	—	5.242	5.729	5.420	3.800

**TABLE 7:** The QSAR model of PTGS1/2 predicts the PIC_50_ value of the compound.

Component	PTGS1	PIC_50_ (μM)	PTGS2	PIC_50_ (μM)
Salvianolic acid B	6.079	0.834	5.490	3.236
Rosmarinic acid	5.946	1.132	4.743	18.071
Lithospermic acid	6.079	0.834	5.404	3.945
Salvianolic acid D	6.568	0.270	5.501	3.155
Salvianolic acid Y	6.079	0.834	5.490	3.236

### Validation of SAFI Targets *in vitro*

**FIGURE 5:**
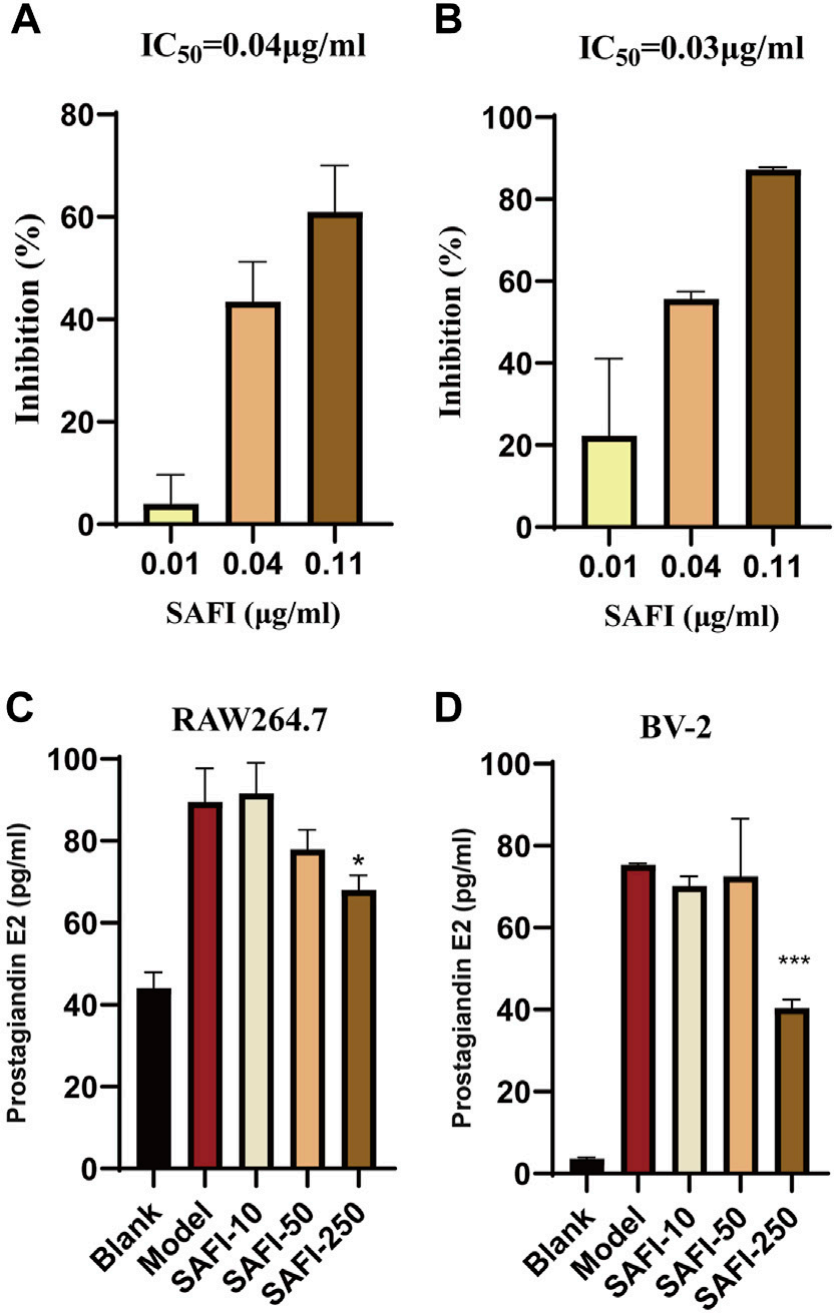
SAFI inhibits PTGS1 and PTGS2. *In vitro* inhibitory of effect of SAFI on PTGS1 **(A)** and PTGS2 **(B)** enzymatic activities. IC_50_ values obtained are indicated on top of histograms. **(C)** SAFI inhibits the synthesis of prostaglandin E2 in RAW264.7 macrophages. Cells were pre-treated with increasing concentrations of SAFI for 1 h and then stimulated with LPS for 18 h to induce prostaglandin E2 expression.**(D)** SAFI inhibits the synthesis of prostaglandin E2 in BV-2 microglia. Cells were treated with LPS and different concentrations of SAFI for 24 h to induce prostaglandin E2 expression. *Blank* indicates cells treated without SAFI or LPS, and *Model* indicates cells treated with LPS alone. Data represent the average ±SEM of three independent replicates.

Arachidonic acid metabolism plays an important role in acute ischemic syndromes affecting the coronary or cerebrovascular territory, whereas, cyclooxygenase, including PTGS1 and PTGS2, is the key enzyme of the arachidonic acid metabolism (Santovito et al., 2009; Cipollone et al., 2010). Our computational prediction results show that SAFI has an inhibitory effect on these two enzymes. To validate the effect of SAFI on PTGS1 and PTGS2, we determined how their enzymatic activity was modulated. The enzymatic assay showed that SAFI had a strong inhibition on PTGS1 and PTGS2, with IC_50_ of 0.04 μg/ml (Figure 5A) and 0.03 μg/ml (Figure 5B) respectively. The inhibition curves of positive controls SC-560 and celecoxib on PTGS1 and PTGS2 can be seen in Supplementary Figure S3. In order to validate the anti-inflammation effect of SAFI, we determined the content of prostaglandin E2 induced by LPS in RAW264.7 macrophages and BV-2 microglia, respectively. As shown in Figure 5C, SAFI, at a concentration of 250 μg/ml, had a significant inhibition on the production of prostaglandin E2 both in the RAW264.7 macrophages and BV-2 microglia. These indicate that SAFI inhibits the production of prostaglandin E2 in a cell-based assay, and these offer a mode of action for its anti-inflammation effect.

## Discussion

### Mechanism of Action of SAFI in IS

Intravenous administration of tissue plasminogen activator and endovascular treatment are currently used to recanalize the blood flow in acute IS. However, reperfusion leads to a highly harmful reactive oxygen species production, generating oxidative stress, which is responsible for most of the ischemia-reperfusion injury and brain tissue damage (Sofía et al., 2020). The positive effect of SAFI on ischemia reperfusion injury has been widely reported in the literature (Tang et al., 2015; Wang et al., 2017; Huang et al., 2019), although its targets and mechanism of action remain to be discovered.

In this study, we have systemically analyzed the mechanism of action of SAFI on IS using the network pharmacology combined computational prediction and experimental validation. We identified 38 genes shared by datasets of SAFI targets, SAFI main components targets and IS-associated genes. GO and KEGG enrichment analysis of this common shared gene set indicates their association with inflammation. Two genes associated with inflammation (*PTGS1* and *PTGS2*) were preliminarily validated *in vitro*. SAFI inhibited PTGS1 and PTGS2 activity in a dose-dependent manner and inhibited the production of prostaglandin E2 induced by LPS in RAW264.7 macrophages and BV-2 microglia. Cytokines, like tumor necrosis factor (TNF), modulate tissue injury in experimental stroke and affect infarct evolution, making them good targets for potential future stroke therapy (Lam Be Rtsen et al., 2012). Nuclear factor kappa B (NF-κB) is a key regulator of the inflammatory process, regulating expression of proinflammatory and proapoptotic genes in ischemia-reperfusion injury of the brain and representing a therapeutic target of IS (Howell and Bidwell, 2020). HIF-1α and Notch-1 engage pro-inflammatory and apoptotic signaling pathways, thus promoting neuronal cell death in IS (Cheng et al., 2014). SAFI and its main component, Salvianolic acid B, have been reported to inhibit IL-1β, IL-6 NF-κB, TNF, and HIF-1 (Ming-chao et al., 2016; Wang et al., 2016; Wang et al., 2017; Zhuang et al., 2017; Fan et al., 2018; Zhao et al., 2020). Interestingly, as one of the main components, no potential targets of salvianolic acid Y were obtained from HERB database and literature mining. The only report related to salvianolic acid Y was about its antioxidant effect (Jun et al., 2015). In view of the similar structure with salvianolic acid B, the potential anti-inflammatory activity of salvianolic acid Y deserves to be mined in the future. QASR model prediction analysis indicates that SAFI has the potential to bind to PTGS1 and PTGS2, with binding as good as the positive drugs, aspirin and NS-398. Drug similarity analysis also indicates that SAFI and aspirin may share a common set of targets. This highlights once more that the effect of SAFI on IS may be due to its anti-inflammatory effect by inhibiting the activity of PTGS1 and/or PTGS2. Aspirin has been frequently prescribed to prevent cardiovascular disease due to its analgesic, anti-pyretic, anti-inflammatory and antithrombotic qualities. However, its side effects, such as internal bleeding and gastrointestinal damage, should also be noted when long-term medication is used (Sostres and Lanas, 2011; Fiala and Pasic, 2020). SAFI has been widely used to treat IS clinically in China, mostly for mild to moderate cases, with no gastrointestinal side effects. Although SAFI has been reported to inhibit the expression level of PTGS2, but not PTGS1, the relationship between SAFI, IS and PTGS1/2 was not confirmed (Wang et al., 2017). Therefore the interaction between SAFI and PTGS1/PTGS2 in IS are still not clear in the current literature. Overall, our network pharmacology approach indicates that the beneficial effect of SAFI on IS is likely due to its anti-inflammatory properties *via* direct inhibition of PTGS1 and PTGS2. It should be noted that PTGS1 and PTGS2 are involved in inflammation not only via the arachidonic acid (AA) metabolic pathway, but also through the maintenance of cerebral blood flow, synaptic plasticity, and cerebrovascular regulation (Niwa et al., 2000; Chen and Bazan, 2005; Jayaraj et al., 2020). Due to the advantages of multiple components, multiple targets and multiple pathways, other potential effects of SAFI in the treatment of IS should be further explored.

### SAFI as a Case Study for the Use of the Computational Systems Pharmacology to Elucidate Mechanisms of TCM

In this study, we have used network pharmacology combined with computational prediction to elucidate the molecular mechanisms of SAFI acting on IS, and demonstrated the feasibility of the approach in investigating its mode of action. A similar approach can be used for a variety of herbal medicine used in TCM for a variety of diseases, thus contributing to its modernization.

The emergence of TCM databases such as HERB, TCMSP and others makes relatively easy to obtain the chemical components and corresponding targets of TCM, saving a lot of time and cost of experiments (Ru et al., 2014; Fang et al., 2020). However, the chemical components in the prescription and their pharmacological characteristics, such as their absorption, have not been fully evaluated in many of them, which may lead to some bias when using enrichment analyses.

Propagation-like algorithms, such as SRWR, have been used in the identification of proteins or genes influenced by drugs or diseases, and can simulate the spread of active compound-induced activation or inhibition to a group of targets on a particular signaling network(Zhao et al., 2019). Using this algorithm, we have identified the top 10% genes inhibited by SAFI, and enriched them by pathways and biological process analysis. The results illustrate the mechanism by which SAFI likely acts when used to treat IS: inhibition of a group of proteins associated with inflammation. However, the human signaling network may not include some unknown protein interactions with orientation and/or patterns, and this may lead to some loss of information in subsequent identification analysis of inhibited and activated genes. Given that, more studies using high-throughput data such as RNA sequencing, single-cell sequencing and other computational models to elucidate the mechanisms of SAFI treating IS are warranted, which may provide us more rich and accurate information on IS(Olsen and Baryawno, 2018; Peng et al., 2021; Zheng et al., 2022).

QSAR has been applied for decades in the development of relationships between physicochemical properties of chemical substances and their biological activities in order to obtain a reliable statistical model for prediction of the activities of new chemical entities (Verma et al., 2010). Since the study of QSAR is based on a series of assumptions using known compounds, this methodology does not consider the possible interactions of complex components in SAFI, or other TCMs, and there may be deviations between predicted activities and actual conditions.

In summary, in this study we deduce the mode of action of SAFI on IS from the levels of the components, targets, pathways, network, and activity prediction. SAFI putative targets are significantly enriched in several pathways associated with inflammation, which is critical in IS. *PTGS1* and *PTGS2* were found in a dataset of genes shared between SAFI and its components’ targets and IS-associated genes, and their binding activities were further predicted by QSAR. The effect of SAFI on the enzymatic activities of PTGS1 and PTGS2 confirmed the data deduced from network pharmacology combined with computational prediction. Due to the limitations of network pharmacology and the complicated pathogenesis progress of IS, more extensive and in-depth research is still needed to corroborate these data to elucidate other action mechanisms of SAFI in the treatment of IS.

## Data Availability

The original contributions presented in the study are included in the article/Supplementary Material, further inquiries can be directed to the corresponding author.

## References

[B1] Anna G. Bellis L. J. Patricia B. A. Jon C. Mark D. Anne H. (2012). ChEMBL: a Large-Scale Bioactivity Database for Drug Discovery. Nucleic acids Res. 40, 1100–1107. 10.1093/nar/gkr777 PMC3245175 21948594

[B2] Barabási A. Gulbahce N. Loscalzo J. (2011). Network Medicine: a Network-Based Approach to Human Disease. Nat. Rev. Genet. 12, 56–68. 21164525 10.1038/nrg2918 PMC3140052

[B3] Chen C. Bazan N. G. (2005). Lipid Signaling: Sleep, Synaptic Plasticity, and Neuroprotection. Prostagl. Other Lipid Mediat 77, 65–76. 10.1016/j.prostaglandins.2005.07.001 16099392

[B4] Cheng Y. L. Park J. S. Manzanero S. Choi Y. Baik S. H. Okun E. (2014). Evidence that Collaboration between HIF-1α and Notch-1 Promotes Neuronal Cell Death in Ischemic Stroke. Neurobiol. Dis. 62, 286–295. 10.1016/j.nbd.2013.10.009 24141018 PMC3877697

[B5] Cipollone F. Fazia M. Mezzetti A. (2010). Novel Determinants of Plaque Instability. J. Thromb. Haemost. 3, 1962–1975. 10.1111/j.1538-7836.2005.01355.x 16102103

[B6] Datta A. Sarmah D. Mounica L. Kaur H. Kesharwani R. Verma G. (2020). Cell Death Pathways in Ischemic Stroke and Targeted Pharmacotherapy. Transl. Stroke Res. 11, 1185–1202. 10.1007/s12975-020-00806-z 32219729

[B7] De Oliveira M. T. Katekawa E. (2018). On the Virtues of Automated Quantitative Structure-Activity Relationship: the New Kid on the Block. Future Med. Chem. 10, 335–342. 10.4155/fmc-2017-0170 29393678

[B8] Dixon S. L. Duan J. Smith E. Von Bargen C. D. Sherman W. Repasky M. P. (2016). AutoQSAR: an Automated Machine Learning Tool for Best-Practice Quantitative Structure-Activity Relationship Modeling. Future Med. Chem. 8, 1825–1839. 10.4155/fmc-2016-0093 27643715

[B9] Du X. Yang J. Liu C. Wang S. Zhang C. Zhao H. (2020). Hypoxia-Inducible Factor 1α and 2α Have Beneficial Effects in Remote Ischemic Preconditioning against Stroke by Modulating Inflammatory Responses in Aged Rats. Front. Aging Neurosci. 12, 54. 10.3389/fnagi.2020.00054 32210788 PMC7076079

[B10] Fan Y. Luo Q. Wei J. Lin R. Lin L. Li Y. (2018). Mechanism of Salvianolic Acid B Neuroprotection against Ischemia/reperfusion Induced Cerebral Injury. Brain Res. 1679, 125–133. 29180227 10.1016/j.brainres.2017.11.027

[B11] Fang S. Dong L. Liu L. Guo J. Zhao L. Zhang J. (2020). HERB: a High-Throughput Experiment- and Reference-Guided Database of Traditional Chinese Medicine. Nucleic Acids Res. 49, 1197–1206. 10.1093/nar/gkaa1063 PMC7779036 33264402

[B12] Fg A. Xuan T. Wen Z. Wei J. Tang S. Wu H. (2020). Exploration of the Mechanism of Traditional Chinese Medicine by AI Approach Using Unsupervised Machine Learning for Cellular Functional Similarity of Compounds in Heterogeneous Networks, XiaoErFuPi Granules as an Example. Pharmacol. Res. 160, 105077. 32687952 10.1016/j.phrs.2020.105077

[B13] Fiala C. Pasic M. D. (2020). Aspirin: Bitter Pill or Miracle Drug? Clin. Biochem. 85, 1–4. 10.1016/j.clinbiochem.2020.07.003 32721423

[B14] Hakime Ö. Elif O. Arzucan Ö. (2016). A Comparative Study of SMILES-Based Compound Similarity Functions for Drug-Target Interaction Prediction. BMC Bioinforma. 17, 128. 10.1186/s12859-016-0977-x PMC4797122 26987649

[B15] Herpich F. Rincon F. (2020). Management of Acute Ischemic Stroke. Crit. Care Med. 48, 1654–1663. 10.1097/CCM.0000000000004597 32947473 PMC7540624

[B16] Howell J. A. Bidwell G. L. (2020). Targeting the NF-Κb Pathway for Therapy of Ischemic Stroke. Ther. Deliv. 11, 113–123. 10.4155/tde-2019-0075 31928138

[B17] Huang D. D. Wei X. H. Mu H. N. Pan C. S. Li Q. Hu B. H. (2019). Total Salvianolic Acid Injection Prevents Ischemia/Reperfusion-Induced Myocardial Injury via Antioxidant Mechanism Involving Mitochondrial Respiratory Chain through the Upregulation of Sirtuin1 and Sirtuin3. Shock 51, 745–756. 10.1097/SHK.0000000000001185 29863652 PMC6511432

[B18] Jayaraj R. L. Azimullah S. Beiram R. Jalal F. Y. Rosenberg G. A. (2020). Neuroinflammation: Friend and Foe for Ischemic Stroke. J. Neuroinflammation 16, 142. 10.1186/s12974-019-1516-2 PMC6617684 31291966

[B19] Jiao X. Jin X. Ma Y. Yang Y. Li J. Liang L. (2021). A Comprehensive Application: Molecular Docking and Network Pharmacology for the Prediction of Bioactive Constituents and Elucidation of Mechanisms of Action in Component-Based Chinese Medicine. Comput. Biol. Chem. 90, 107402. 10.1016/j.compbiolchem.2020.107402 33338839

[B20] Jing X. Zheng-Liang Y. De-Kun L. Da-Zheng Z. Bing L. (2013). Simultaneous Determination of Salvianolic Acid DRosmarinic AcidLithospermic Acid and Salvianolic Acid B in the Salvianolic Acid Extract by HPLC. Chin. J. Exp. Traditional Med. Formulae 19, 70–73.

[B21] Jing-Yao X. Xiao-Lin L. Ling T. Hong-Shui Y. Ai-Chun J. Zhi-Guo Y. (2015). HPLC Determination of Six Water-Soluble Chemical Constituents in Salvianolic Acid for Injection. Chin. J. New Drugs 24, 1599–1603.

[B22] Jun G. Aichun J. Dazheng Z. Dekun L. Wei Z. Wanli G. (2015). Salvianolic Acid Y: A New Protector of PC12 Cells against Hydrogen Peroxide-Induced Injury from Salvia Officinalis. Molecules 20, 683–692. 10.3390/molecules20010683 25569522 PMC6272257

[B23] Jung J. Jin W. Sael L. Kang U. (2016). “Personalized Ranking in Signed Networks Using Signed Random Walk with Restart,” in IEEE 16th International Conference On Data Mining (ICDM), Barcelona, Spain, 12-15 Dec, 2016. 10.1109/icdm.2016.0122

[B24] Köhler S. Bauer S. Horn D. Robinson P. N. (2008). Walking the Interactome for Prioritization of Candidate Disease Genes. Am. J. Hum. Genet. 82, 949–958. 10.1016/j.ajhg.2008.02.013 18371930 PMC2427257

[B25] Lam Be Rtsen K. L. Bi Be. R. K. Finsen B. (2012). Inflammatory Cytokines in Experimental and Human Stroke. Cereb. Blood Flow. Metab. 32, 1677–1698. 10.1038/jcbfm.2012.88 PMC3434626 22739623

[B26] Lei X. Shiyuan W. Yuefei W. Meng W. Guixiang P. Miaomiao J. (2015). Simultaneous Determination of Rosmarinic Acid, Lithospermic Acid, Salvianolic Acid B and Mannitol in Salvianolate Lyophilized Injection by NMR. Chin. Tradit. Pat. Med. 37, 2185–2189.

[B27] Li L. Xiangmei H. Jun G. Dekun L. Dazheng Z. Yuewu Y. (2016). Simultaneous Determination of Salvianolic Acid Y and the Other 3 Components in Salvianolic Acid Extract by HPLC-UV. J. AOAC. Int. 18, 35–37. 10.5740/jaoacint.11-234

[B28] Li S. Zhang B. (2013). Traditional Chinese Medicine Network Pharmacology: Theory, Methodology and Application. Chin. J. Nat. Med. 11, 110–120. 10.1016/S1875-5364(13)60037-0 23787177

[B29] Li W. Polachi N. Wang X. Chu Y. Wang Y. Tian M. (2018). A Quality Marker Study on Salvianolic Acids for Injection. Phytomedicine 44, 138–147. 10.1016/j.phymed.2018.02.003 29544864

[B30] Maida C. D. Norrito R. L. Daidone M. Tuttolomondo A. Pinto A. (2020). Neuroinflammatory Mechanisms in Ischemic Stroke: Focus on Cardioembolic Stroke, Background, and Therapeutic Approaches. Int. J. Mol. Sci. 21, 1–33. 10.3390/ijms21186454 PMC7555650 32899616

[B31] Menche J. Sharma A. Kitsak M. Ghiassian S. D. Vidal M. Loscalzo J. (2015). Disease Networks. Uncovering Disease-Disease Relationships through the Incomplete Interactome. Science 347, 1257601. 10.1126/science.1257601 25700523 PMC4435741

[B32] Ming-Chao Y. You F-L. Wang Z. Liu X-N. Wang Y-F. (2016). Salvianolic Acid B Improves the Disruption of High Glucose-Mediated Brain Microvascular Endothelial Cells via the ROS/HIF-1α/VEGF and miR-200b/VEGF Signaling Pathways. Neurosci. Lett. 630, 233–240. 10.1016/j.neulet.2016.08.005 27497919

[B33] Minghua X. Jiale C. Kening Z. Qu L. Yali L. Huiting L. (2021). Aloe-emodin Prevents Nerve Injury and Neuroinflammation Caused by Ischemic Stroke via the PI3K/AKT/mTOR and NF-Κb Pathway. Food & Funct. 12, 8056–8067. 10.1039/d1fo01144h 34286782

[B34] Neurology C. S. O. Society C. S. (2018). Chinese Guidelines for Diagnosis and Treatment of Acute Ischemic Stroke 2018. Chin. J. Neurology 51, 666–682.

[B35] Niwa K. Araki E. Morham S. G. Ross M. E. Iadecola C. (2000). Cyclooxygenase-2 Contributes to Functional Hyperemia in Whisker-Barrel Cortex. J. Neurosci. 20, 763–770. 10.1523/jneurosci.20-02-00763.2000 10632605 PMC6772412

[B36] Olsen T. K. Baryawno N. (2018). Introduction to Single-Cell RNA Sequencing. Curr. Protoc. Mol. Biol. 122, e57. 10.1002/cpmb.57 29851283

[B37] Peng J. Q. Ren J. G. Liu J. X. (2021). Application Prospects of Single-Cell Transcriptome Sequencing in Traditional Chinese Medicine Research. Zhongguo Zhong Yao Za Zhi 46, 2456–2460. 10.19540/j.cnki.cjcmm.20210218.601 34047090

[B38] Piero J. Ramírez-Anguita J. Saüch-Pitarch J. Ronzano F. Furlong L. I. (2019). The DisGeNET Knowledge Platform for Disease Genomics: 2019 Update. Nucleic Acids Res. 48. 10.1093/nar/gkz1021 PMC7145631 31680165

[B39] Ru J. Li P. Wang J. Zhou W. Li B. Huang C. (2014). TCMSP: a Database of Systems Pharmacology for Drug Discovery from Herbal Medicines. J. Cheminform 6, 13. 10.1186/1758-2946-6-13 24735618 PMC4001360

[B40] Santovito D. Mezzetti A. Cipollone F. (2009). Cyclooxygenase and Prostaglandin Synthases: Roles in Plaque Stability and Instability in Humans. Curr. Opin. Lipidol. 20, 402–408. 10.1097/MOL.0b013e32832fa22c 19741338

[B41] Sofía O.-U. Ignacio R. Lucas L. Ramón R. (2020). Pathophysiology of Ischemic Stroke: Role of Oxidative Stress. Curr. Pharm. Des. 26, 4246–4260. 32640953 10.2174/1381612826666200708133912

[B42] Sostres C. Lanas A. (2011). Gastrointestinal Effects of Aspirin. Nat. Rev. Gastroenterol. Hepatol. 8, 385–394. 10.1038/nrgastro.2011.97 21647198

[B44] Sun Y. Han J. Yan X. Yu P. S. Wu T. (2011). PathSim: Meta Path-Based Top-K Similarity Search in Heterogeneous Information Networks. J Proc. Vldb Endow. 4, 992–1003.

[B45] Tang H. Pan C. S. Mao X. W. Liu Y. Y. Yan L. Zhou C. M. (2015). Role of NADPH Oxidase in Total Salvianolic Acid Injection Attenuating Ischemia‐Reperfusion Impaired Cerebral Microcirculation and Neurons: Implication of AMPK/Akt/PKC. Microcirculation 21, 615–627. 10.1111/micc.12140 24702968

[B46] The Gene Ontology C. (2017). Expansion of the Gene Ontology Knowledgebase and Resources. Nucleic Acids Res. 45, D331–D338. 10.1093/nar/gkw1108 27899567 PMC5210579

[B47] Verma J. Khedkar V. M. Coutinho E. C. (2010). 3D-QSAR in Drug Design-Aa Review. Curr. Top. Med. Chem. 10, 95–115. 10.2174/156802610790232260 19929826

[B48] Wang F. He Q. Wang J. Yuan Q. Guo H. Chai L. (2017). Neuroprotective Effect of Salvianolate Lyophilized Injection against Cerebral Ischemia in Type 1 Diabetic Rats. BMC Complement. Altern. Med. 17, 258. 10.1186/s12906-017-1738-8 28486941 PMC5424323

[B49] Wang X. Wang Z. Y. Zheng J. H. Li S. (2021). TCM Network Pharmacology: A New Trend towards Combining Computational, Experimental and Clinical Approaches. Chin. J. Nat. Med. 19, 1–11. 10.1016/s1875-5364(21)60001-8 33516447

[B50] Wang Y. Chen G. Yu X. Li Y. Zhang L. He Z. (2016). Salvianolic Acid B Ameliorates Cerebral Ischemia/Reperfusion Injury through Inhibiting TLR4/MyD88 Signaling Pathway. Inflammation 39, 1503–1513. 10.1007/s10753-016-0384-5 27255374

[B51] Wishart D. S. Feunang Y. D. An C. G. Lo E. J. Wilson M. (2017). DrugBank 5.0: A Major Update to the DrugBank Database for 2018. Nucleic Acids Res. 46. 10.1093/nar/gkx1037 PMC5753335 29126136

[B52] Yang J. Tian S. Zhao J. Zhang W. (2020). Exploring the Mechanism of TCM Formulae in the Treatment of Different Types of Coronary Heart Disease by Network Pharmacology and Machining Learning. Pharmacol. Res. 159, 105034. 10.1016/j.phrs.2020.105034 32565312

[B53] Yang N. (2020). Protective Effect and Mechanism Study of Salvianolate Injection in the Treatment of Acute Ischemic Stroke. Doctor DOCTORAL DISSERTATION. Tianjin, China: Tianjin medical university.

[B54] Zhang W. Huai Y. Miao Z. Qian A. Wang Y. (2019). Systems Pharmacology for Investigation of the Mechanisms of Action of Traditional Chinese Medicine in Drug Discovery. Front. Pharmacol. 10, 743. 10.3389/fphar.2019.00743 31379563 PMC6657703

[B55] Zhao J. Chao L. Qiu-Ling W. U. Zeng H. W. Guo X. Yang J. (2019). Computational Systems Pharmacology Reveals Anantiplatelet and Neuroprotective Mechanism of Deng-Zhan-Xi-Xin Injection in Treatment of Ischemic Stroke. Pharmacol. Res. 147. 10.1016/j.phrs.2019.104365 31348992

[B56] Zhao M. Li F. Jian Y. Wang X. Yang H. Wang J. (2020). Salvianolic Acid B Regulates Macrophage Polarization in Ischemic/reperfused Hearts by Inhibiting mTORC1-Induced Glycolysis. Eur. J. Pharmacol. 871, 172916. 10.1016/j.ejphar.2020.172916 31930970

[B57] Zheng K. Lin L. Jiang W. Chen L. Zhang X. Zhang Q. (2022). Single-cell RNA-Seq Reveals the Transcriptional Landscape in Ischemic Stroke. J. Cereb. Blood Flow. Metab. 42, 56–73. 10.1177/0271678X211026770 34496660 PMC8721774

[B58] Zhuang P. Wan Y. Geng S. He Y. Feng B. Ye Z. (2017). Salvianolic Acids for Injection (SAFI) Suppresses Inflammatory Responses in Activated Microglia to Attenuate Brain Damage in Focal Cerebral Ischemia. J. Ethnopharmacol. 198, 194–204. 10.1016/j.jep.2016.11.052 28087473

