# Supplementary figures and images for: Exploration of the Mechanism of Salvianolic Acid for Injection Against Ischemic Stroke: A Research Based on Computational Prediction and Experimental Validation
Source: Front Pharmacol. 2022 May 25;13:894427. doi: 10.3389/fphar.2022.894427 (PMC9175744; doi:10.3389/fphar.2022.894427)

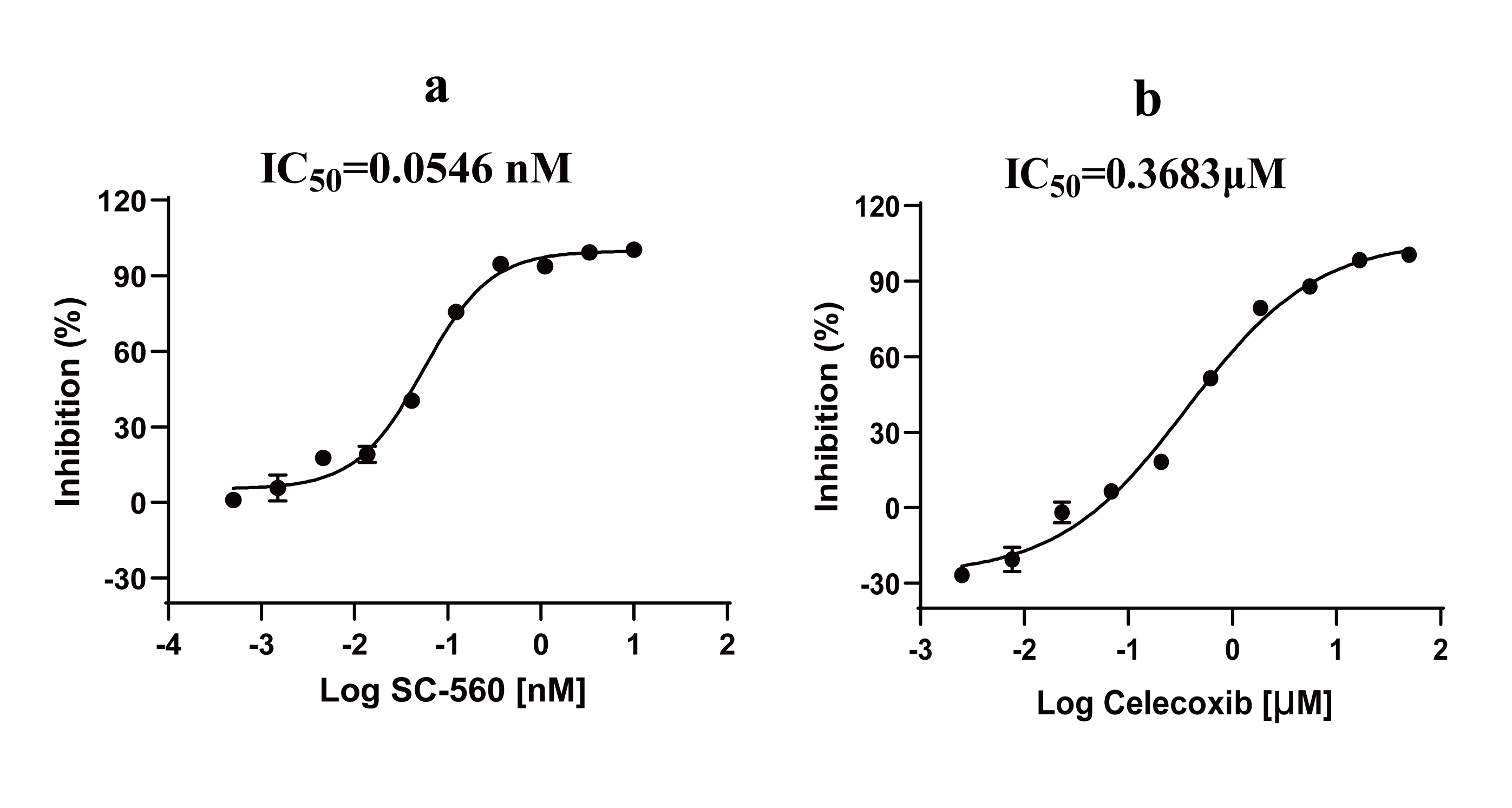

Supplement: Supplementary file 2 [file Image3.JPEG]

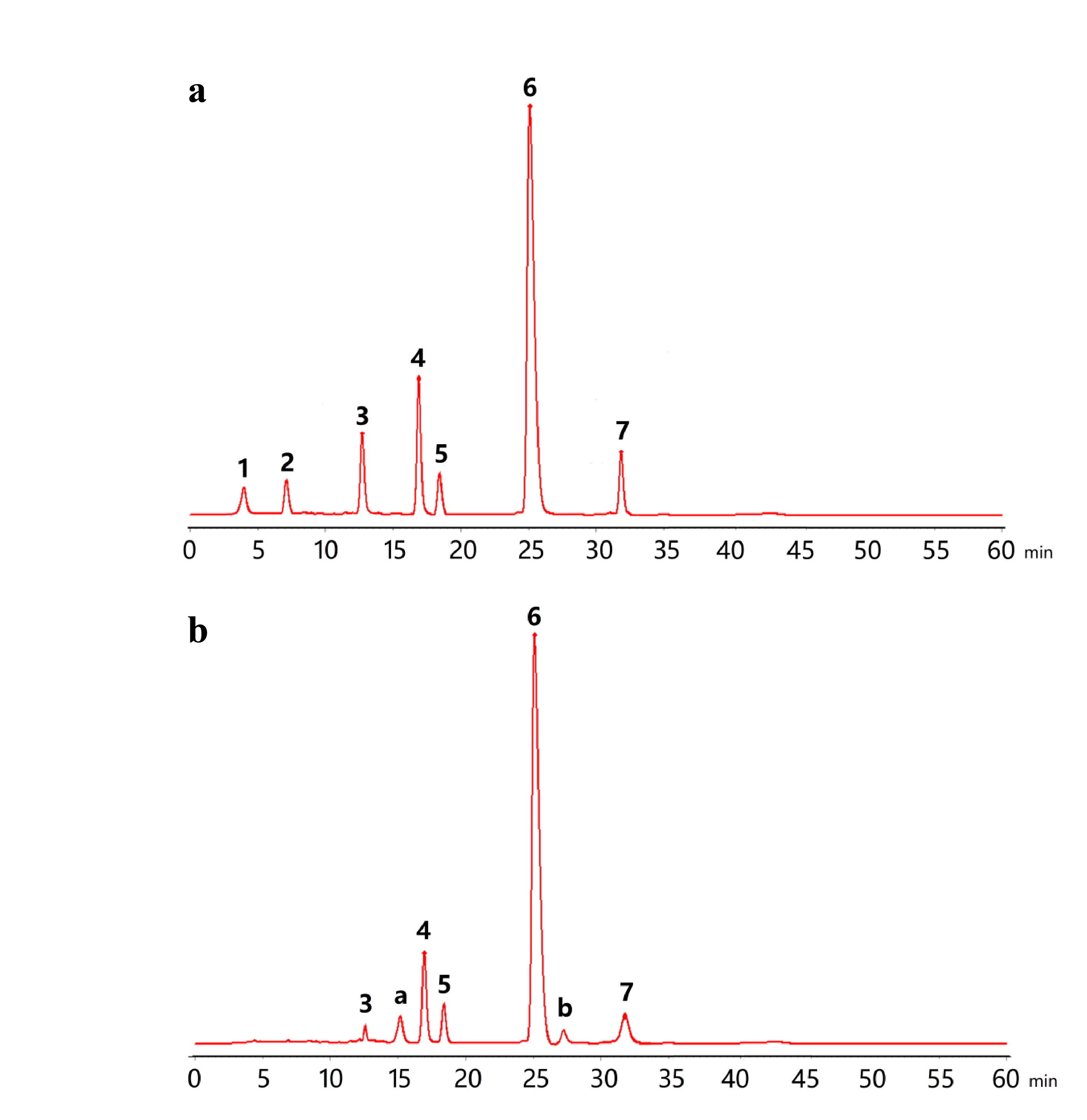

Supplement: Supplementary file 3 [file Image1.JPEG]

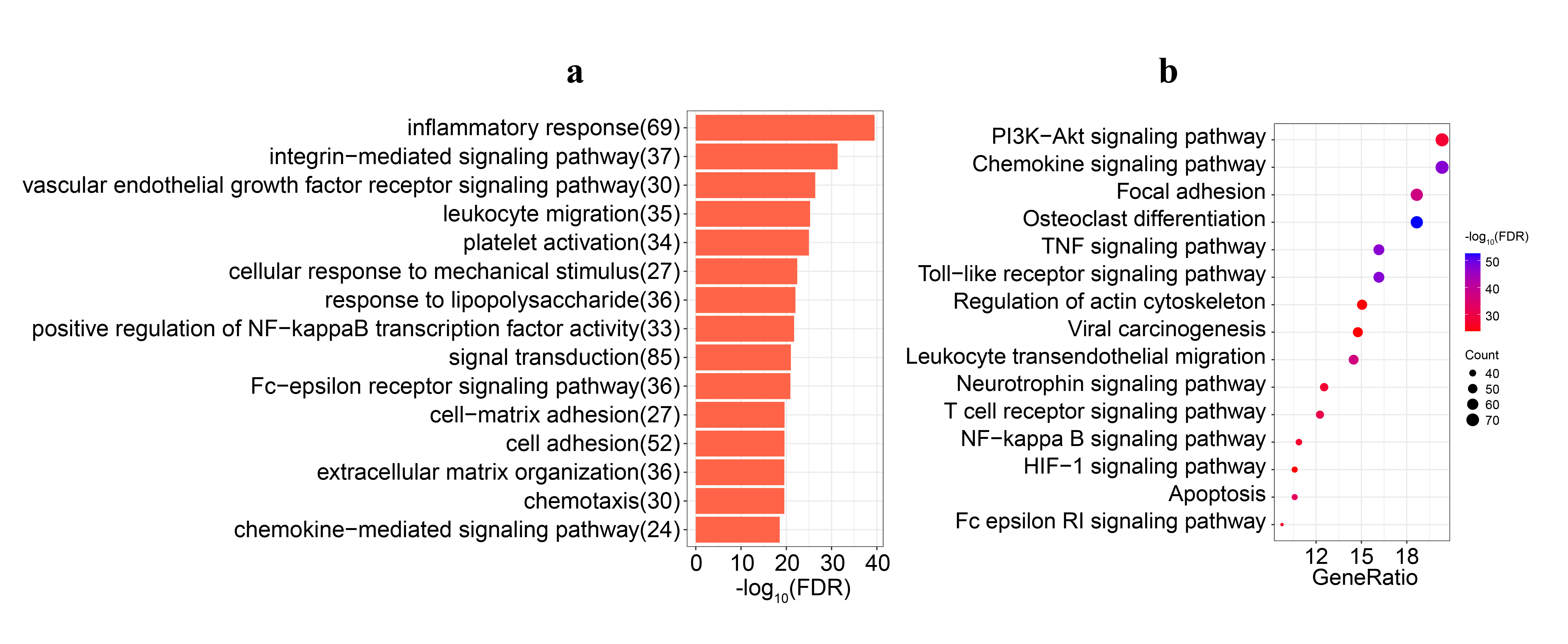

Supplement: Supplementary file 4 [file Image2.JPEG]
