# Supplementary material for: Exploration of the Mechanism of Salvianolic Acid for Injection Against Ischemic Stroke: A Research Based on Computational Prediction and Experimental Validation
Source: Front Pharmacol. 2022 May 25;13:894427. doi: 10.3389/fphar.2022.894427 (PMC9175744; doi:10.3389/fphar.2022.894427)
Supplement: Supplementary file 5 [file DataSheet1.pdf]

Figure 1. Workflow illustrating the steps used in the elucidation of SAFI mechanism of action in IS treatment. The workflow includes data collection, data analysis and experimental validations.

Figure 2. Potential targets of SAFI in the treatment of IS.
(A) The main components and contents in SAFI collected from literature mining. It has to be noted that mannitol is used as pharmaceutic adjuvant in SAFI. (B) Potential targets of main components in SAFI. Pink boxes represent salvianolic acid B, rosmarinic acid, lithopermic acid and salvianolic acid D, and green rounded boxes represent the corresponding targets of different components of SAFI collected from HERB database.(C) Overlaps between IS-associated genes from DisGeNET database, targets of SAFI collected from literature mining and targets of main components of SAFI (salvianolic acid B, rosmarinic acid, lithopermic acid, and salvianolic acid D) collected from literature mining and HERB database. (D,E) Functional annotations (biological process,D, and pathways, E) of 38 common genes obtained from overlaps by enrichment analysis.

Figure 3. Drug similarity based on structures, functions and targets. Salvianolic acid B, rosmarinic acid, lithopermic acid, salvianolic acid Y, salvianolic acid D were compared with 16 recommended anti-IS drugs based on chemical structures (A), functions (B) and targets (C). In A and B panels, all compounds were divided into 6 subclusters with different background colors based on hierarchical clustering. Different types of approved drugs against IS are marked with different colors. In panel C, the depth of color indicates the network proximity ($S_{AB}$) of the two drugs. The closer the color is getting to red, the smaller the $S_{AB}$, and thus, the closer the topological distance between the two drugs, that is, the more similar the targets of the two drugs.

Figure 4. Scatter plots illustrating the performance of the QSAR model in predicting the experimental binding affinity of the learning set. (A) Model code: kpls_radial_36, generated by KPLS fitting radical features, using the 36th split of the learning set into test and training sets; (B) Model code: kpls_linear_36, generated by KPLS fitting linear features, using the 36th split of the learning set into test and training sets; (C) Model code: kpls_linear_46, generated by KPLS fitting linear features, using the 46th split of the learning set into test and training sets; (D) Model code: kpls_radial_46, generated by KPLS fitting radial features, using the 46th split of the learning set into test and training sets.

Figure 5. SAFI inhibits PTGS1 and PTGS2. *In vitro* inhibitory of effect of SAFI on PTGS1 (A) and PTGS2 (B) enzymatic activities. $IC_{50}$ values obtained are indicated on top of histograms. (C) SAFI inhibits the synthesis of prostaglandin E2 in RAW264.7 macrophages. Cells were pre-treated with increasing concentrations of SAFI for 1 h and then stimulated with LPS for 18 h to induce prostaglandin E2 expression.(D) SAFI inhibits the synthesis of prostaglandin E2 in BV-2 microglia. Cells were treated with LPS and different concentrations of SAFI for 24h to induce prostaglandin E2 expression. *Blank* indicates cells treated without SAFI or LPS, and *Model* indicates cells treated with LPS alone. Data represent the average ± SEM of three independent replicates.

Supplementary Figure 1. Representative HPLC chromatograms for the determination of Q-Markers in SAFI. (A) The reference compound mixture (B) SAFI. 1: Danshensu, 2: Protocatechuic aldehyde, 3: Salvianolic acid D, 4: Rosmarinic acid, 5: Lithospermic acid, 6: Salvianolic acid B, 7: Salvianolic acid Y, a: Salvianolic acid B isomer, b: Salvianolic acid B isomer. The figure is taken from Li, W., et al. (2018).

Supplementary Figure 2. Functional annotations of 362 top inhibited genes by enrichment analysis. Biological process (A) and pathways (B) were enriched with 362 genes predicted by SRWR algorithm.

Supplementary Figure 3. Inhibition curves of positive controls SC-560 on PTGS1 (A) and celecoxib on PTGS2 (B) enzymatic activities. The $IC_{50}$ obtained are indicated on top of each curve.
